# Age‐Specific Temporal Trends and Climate‐Associated Seasonality of Appendectomy in Japan: A Nationwide Population‐Based Study Using the National Database

**DOI:** 10.1002/ags3.70305

**Published:** 2026-10-30

**Authors:** Shutaro Sumiyoshi, Masamitsu Kido, Tomohiro Arita, Naosuke Nakamichi, Kento Kurashima, Kenji Nanishi, Jun Kiuchi, Hiroki Shimizu, Kengo Yoshii, Atsushi Shiozaki

**Affiliations:** ^1^ Department of Surgery, Division of Digestive Surgery, Graduate School of Medical Science Kyoto Prefectural University of Medicine Kyoto Japan; ^2^ Department of Orthopedic Surgery Inage Hospital Chiba Japan; ^3^ Department of Mathematics and Statistics in Medical Sciences Kyoto Prefectural University of Medicine Kyoto Japan

**Keywords:** appendicitis, Japan, laparoscopic surgery, national database, surgical epidemiology

## Abstract

**Aim:**

This study investigated nationwide temporal trends, age‐specific changes, and seasonal variations in appendectomies in Japan using the national database of health insurance claims and specific health checkups (NDB).

**Methods:**

Data on appendectomies (open [OA] and laparoscopic [LA]) were extracted from the 2018–2023 NDB Open Data. Crude and age‐adjusted appendectomy rates per 100,000 person‐years were calculated. Temporal trends were assessed using Poisson regression, and age‐specific annual relative risks were estimated. Seasonality (April 2019 to March 2024) was evaluated, and correlations with meteorological variables were assessed using Pearson correlation coefficients. Time‐series Poisson regression was used to evaluate the association between monthly appendectomy volume and ambient temperature.

**Results:**

During the study period, 323,980 appendectomies were performed. The age‐adjusted appendectomy rate declined slightly overall (relative risk [RR] = 0.991, *p* < 0.0001), with a significant decrease in men (RR = 0.982, *p* < 0.0001), whereas no significant change was observed in women (RR = 1.003, *p* = 0.0676). Age‐specific analyses demonstrated declining appendectomy rates among younger populations, whereas the overall rates increased after approximately 40 years of age, with larger increases observed among women than men. OA use decreased (RR = 0.793, *p* < 0.0001), whereas LA use increased (RR = 1.022, *p* < 0.0001). The monthly appendectomy volume exhibited significant seasonality and was positively correlated with ambient temperature using meteorological data from five cities.

**Conclusion:**

Appendectomy rates decreased in younger populations but increased among older adults, particularly women. A shift toward laparoscopic surgery and temperature‐associated seasonality was also observed.

## Abstract 2

Using Japan's national database open data from 2018 to 2023, this nationwide study examined temporal trends and seasonal variation in appendectomy rates by age, sex, and surgical approach. Overall appendectomy rates declined, particularly among younger individuals, whereas rates increased among older women; laparoscopic appendectomy also became increasingly predominant. Monthly appendectomy volume was positively associated with mean ambient temperature, demonstrating seasonal variation in appendectomy rates in Japan.

## 1 Introduction

Acute appendicitis is a common gastrointestinal emergency worldwide [1, 2]. Over 20 years, the management of acute appendicitis has evolved considerably with widespread adoption of laparoscopic appendectomy (LA) and increasing acceptance of non‐operative management (NOM) with antibiotics for uncomplicated disease [3, 4, 5]. However, the influence of these evolving treatment strategies on population‐level appendectomy trends across age groups remains unclear.

Appendectomy remains a major treatment option for acute appendicitis, and its utilization may be influenced by both the occurrence of acute appendicitis and changes in treatment strategies. The occurrence of acute appendicitis varies according to age, sex, season, and meteorological conditions, including ambient temperature [1, 6, 7]. Although large‐scale or longitudinal datasets have investigated these associations from Western countries, previous studies in Japan have been limited to smaller, regional, or age‐restricted cohorts [8]. Consequently, nationwide appendectomy trends in aging societies remain unclear. Moreover, aggregated nationwide estimates may mask the heterogeneous temporal trends across demographic subgroups.

Japan offers a unique setting for evaluating population‐based appendectomy epidemiology as among the world's most rapidly aging societies with a universal healthcare system. The national database of health insurance claims and specific health checkups (NDB) accounts for > 95% of insured medical care nationwide [9]. Furthermore, the database allows to evaluate temporal trends, operative approaches, and seasonality at the population level, with minimal selection bias [10, 11].

Using the 2018–2023 Japanese NDB Open Data, this nationwide population‐based study aimed to (1) characterize nationwide temporal trends in appendectomy overall and according to age and sex, (2) evaluate temporal changes in the operative approach from open appendectomy (OA) to LA, and (3) examine the associations between monthly appendectomy volume and meteorological variables from five Japanese cities.

## 2 Methods

### 2.1 Study Design and Data Sources

This nationwide retrospective observational study was conducted using the NDB Open Data, which is publicly released annually by the Ministry of Health, Labour and Welfare of Japan [12]. The NDB is among the largest administrative healthcare databases worldwide, containing reimbursement claims covering > 95% of the Japanese population. The NDB Open Data provide anonymized aggregated procedure counts according to age, sex, and fiscal year. To protect patient privacy, procedure counts < 10 within any stratified category were masked before public release. Appendectomy procedures performed from 2018 to 2023 were analyzed. Annual population estimates for each age and sex group were obtained from official Japanese population statistics (e‐Stat, Statistics Bureau of Japan) [13]. Because this study used publicly available, anonymized, and aggregated data from the NDB Open Data, institutional review board approval and informed consent were not required under the Ethical Guidelines for Life Science and Medical Research Involving Human Subjects.

### 2.2 Selection Criteria for Appendectomy and Classification

Appendectomy procedures were identified using the corresponding Japanese national reimbursement procedure codes: K718 for open appendectomy (OA) and K718‐2 for laparoscopic appendectomy (LA). Accordingly, the analysis was restricted to procedures recorded under these two codes. Cases in which appendicitis was treated with an extended resection beyond appendectomy, such as cecectomy or ileocecal resection, may not have been captured if the procedure was recorded under a different surgical code. Because the NDB Open Data provide aggregated procedure counts without patient‐level clinical information, emergency appendectomy and interval appendectomy following initial NOM could not be distinguished. Similarly, because the NDB Open Data do not contain clinical information regarding disease severity, appendectomies performed for uncomplicated and complicated appendicitis could not be distinguished and were therefore analyzed together.

### 2.3 Outcome Measures

The primary outcome was the temporal trend in nationwide appendectomy rates overall and according to age and sex. Secondary outcomes included temporal changes in operative approach, seasonal variation in monthly appendectomy volume, and the association between appendectomy rates and ambient temperature.

### 2.4 Meteorological Data

Monthly nationwide appendectomy counts from April 2019 to March 2024 were standardized to a 30‐day month to consider differences in month length for graphical presentation, following previously reported methods [14]. Seasons were defined according to the Japan Meteorological Agency as Spring (March–May), Summer (June–August), Autumn (September–November), and Winter (December–February) [15]. Because aggregated nationwide meteorological data were unavailable, monthly mean temperature (°C), mean barometric pressure (hPa), relative humidity (%), daylight hours (h), and total precipitation (mm), were obtained from five representative Japanese cities (Sapporo, Tokyo, Nagoya, Osaka, and Fukuoka) to represent diverse climatic regions across Japan [16]. Given the consistent association between ambient temperature and appendicitis reported in previous epidemiological studies, mean monthly temperature was selected as the primary meteorological variable for the time‐series analysis [6].

### 2.5 Statistical Analysis

Annual crude and age‐adjusted appendectomy rates were calculated per 100,000 person‐years. Crude rates were calculated using the corresponding population as the denominator, based on annual population estimates as of October 1, obtained from e‐Stat [13]. The study included the entire population of Japan, without age restrictions, corresponding to approximately 750 million person‐years over the six fiscal years (2018–2023). Age‐adjusted rates were calculated using the direct method with the 2015 Japanese standard population [11]. Temporal trends in annual appendectomy rates were evaluated using Poisson regression models, with fiscal year entered as a continuous explanatory variable and the natural logarithm of person‐years incorporated as an offset term. For the overall analysis, age group and sex were included as covariates to estimate adjusted annual relative risks (RRs) with 95% confidence intervals (CIs). Interactions between fiscal year and age group and between fiscal year and sex were additionally evaluated to assess differences in temporal trends across age groups and between sexes.

Age‐ and sex‐specific temporal trends were further evaluated using separate Poisson regression models with the natural logarithm of person‐years as an offset. These subgroup analyses were considered exploratory, and no adjustment for multiple comparisons was applied. As a sensitivity analysis, fiscal year was entered as a categorical variable, with fiscal year 2018 as the reference, for the overall population and separately for patients aged < 20, 20–64, and ≥ 65 years. The overall analysis was unadjusted for age and sex, and the age‐specific analyses were conducted separately for each age group.

Monthly seasonal variation in appendectomy volume was evaluated using a time‐series Poisson regression model with monthly appendectomy counts as the dependent variable. The natural logarithm of person‐time (population × number of days in each month) was included as an offset term. Seasonal variation was assessed using a model including season as a categorical variable and calendar time. Associations between monthly appendectomy volume and ambient temperature were evaluated using separate time‐series Poisson regression models for Sapporo, Tokyo, Nagoya, Osaka, and Fukuoka. The same nationwide monthly appendectomy counts were used in each model, whereas the corresponding city‐specific meteorological data were used as the exposure variables. Each model included calendar time, seasonal harmonic terms (sine and cosine functions with a 12 month period), and mean monthly temperature. Mean monthly temperature was selected as the primary meteorological variable based on previous epidemiological evidence [6].

For graphical presentation and Pearson correlation analyses, monthly appendectomy counts standardized to a 30‐day month were used. Pearson correlation coefficients between monthly appendectomy volume and multiple meteorological variables were additionally calculated as exploratory analyses and are presented in Table S2. Statistical significance was defined as a two‐sided *p* < 0.05. Statistical analyses were performed using JMP, version 10 (SAS Institute Inc., Cary, NC, USA).

## 3 Results

### 3.1 Overview

Between 2018 and 2023, 323,980 appendectomies were performed across Japan at a crude rate of 43.0 per 100,000 person‐years. The overall man‐to‐woman ratio was 1.30:1. Age‐stratified analysis revealed a prominent peak in appendectomy rates among the young population aged 10–19 years (Figure 1). Overall, 80.5% of all surgeries were performed in patients aged < 65 years.

**FIGURE 1:**
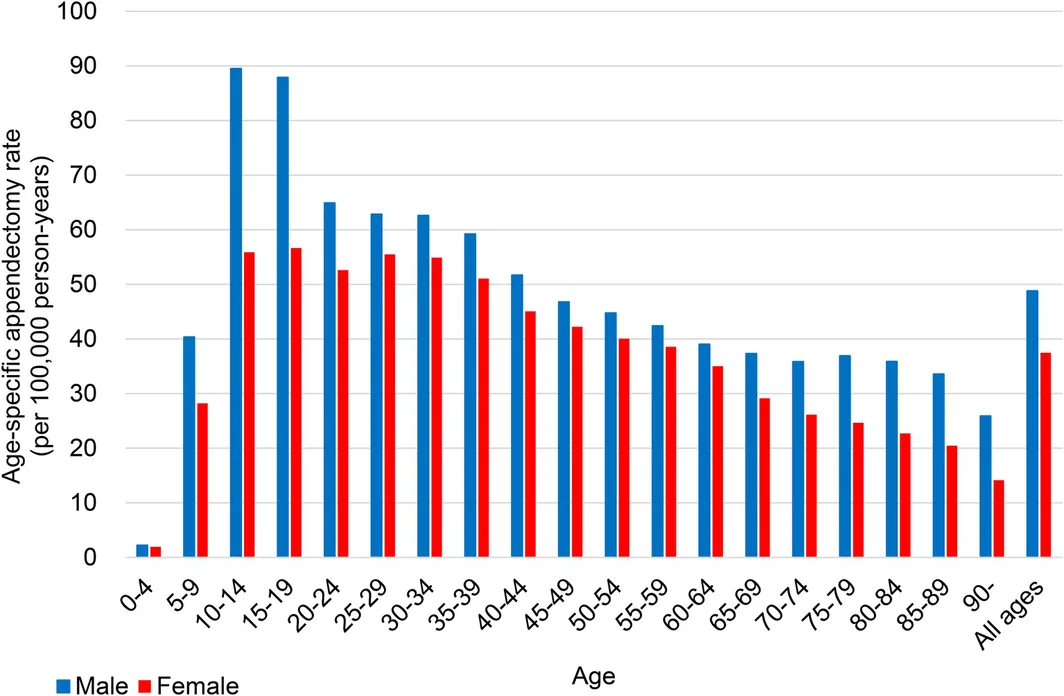
Age‐specific appendectomy rates per 100,000 person‐years in men and women. Overall appendectomy rates were highest among adolescents and young adults, with a secondary peak observed in older adults. Age‐specific temporal changes differ substantially between younger and older populations.

The overall appendectomy rate remained relatively stable during the study period (Figure 2A). However, age‐adjusted appendectomy rates exhibited contrasting temporal patterns. The rate among pediatric and adolescent patients (< 20 years) decreased continuously from 53.9 to 40.8 per 100,000 person‐years between 2018 and 2023. In contrast, the rate among adults aged 20–64 years remained relatively stable (47.5 to 49.0 per 100,000 person‐years), while the rate among older adults aged ≥ 65 years gradually increased from 29.0 to 31.3 per 100,000 person‐years.

**FIGURE 2:**
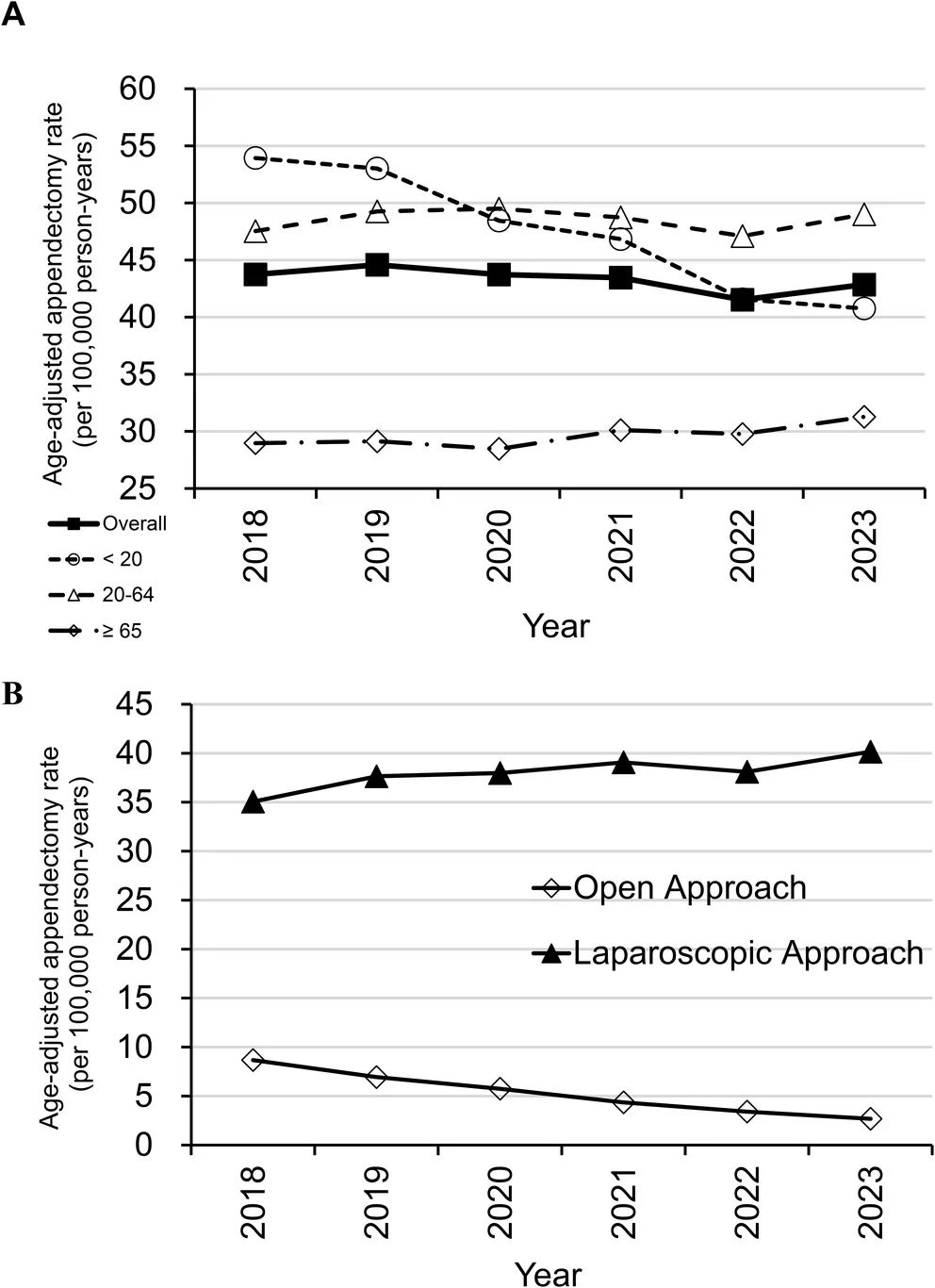
(A) Annual age‐adjusted appendectomy rates overall and according to age category (< 20, 20–64, and ≥ 65 years) from 2018 to 2023. (B) Annual age‐adjusted rates of OA and LA. Age‐adjusted rates were calculated using the 2015 Japanese standard population. OA decreased continuously, whereas LA increased steadily, establishing LA as the predominant operative approach. LA, laparoscopic appendectomy; OA, open appendectomy.

Regarding surgical approaches, a marked and rapid transition from open to laparoscopic procedures was observed nationwide (Figure 2B). The age‐adjusted rate of open appendectomy decreased substantially from 8.68 to 2.69 per 100,000 person‐years between 2018 and 2023, whereas the age‐adjusted rate of laparoscopic appendectomy increased from 35.07 to 40.17 per 100,000 person‐years. Consequently, the proportion of laparoscopic procedures among all appendectomies increased from 80.2% in 2018 to 93.7% in 2023, establishing laparoscopic surgery as the predominant surgical approach in Japan.

### 3.2 Annual Trend Analysis

#### 3.2.1 Annual Trends in Procedure Rates

The overall age‐adjusted appendectomy rate exhibited a slight but statistically significant decline (RR = 0.991, 95% CI; 0.989–0.993, *p* < 0.0001) (Table 1). In absolute terms, the crude appendectomy rate decreased from 43.52 per 100 000 person‐years in 2018 to 42.49 per 100,000 person‐years in 2023, corresponding to an absolute decrease of 1.03 per 100,000 person‐years. The corresponding number of appendectomies decreased from 55,029 to 52,837, a reduction of 2192 procedures (4.0%). A significant interaction was observed between year and sex (*p* for interaction < 0.0001), indicating that temporal trends in appendectomy rates differed between men and women. This nationwide trend was primarily driven by a significant decrease among men (RR = 0.982, 95% CI; 0.979–0.985, *p* < 0.0001), whereas no significant temporal change was observed among women (RR = 1.003, 95% CI; 0.999–1.006, *p* = 0.0676). Concurrently, a marked shift in the surgical approach was evident in age‐adjusted rates. The rate of OA decreased continuously (RR = 0.793, 95% CI; 0.788–0.798, *p* < 0.0001, men: RR = 0.790, women: RR = 0.797, both *p* < 0.0001), whereas the rate of LA increased steadily (RR = 1.022, 95% CI; 1.020–1.024, *p* < 0.0001). The increase in LA rates differed significantly by sex, as indicated by a significant year‐by‐sex interaction (*p* for interaction < 0.0001), with a greater annual increase in women than in men (RR = 1.033 vs. 1.013, 95% CI; 1.030–1.037 vs. 1.010–1.016, respectively, both *p* < 0.0001). A significant interaction was also observed between year and age group (*p* for interaction < 0.0001), indicating that temporal trends differed across age groups.

**TABLE 1:** Poisson regression analysis of annual trends in age‐adjusted appendectomy rates across all ages per 100,000 person‐years.

Variable	RR	95% CI (low)	95% CI (high)	*p*
Overall				
Male	0.982	0.979	0.985	< 0.0001
Female	1.003	0.999	1.006	0.0676
Male & Female	0.991	0.989	0.993	< 0.0001
Open surgery				
Male	0.790	0.784	0.797	< 0.0001
Female	0.797	0.789	0.804	< 0.0001
Male & Female	0.793	0.788	0.798	< 0.0001
Laparoscopic surgery				
Male	1.013	1.010	1.016	< 0.0001
Female	1.033	1.030	1.037	< 0.0001
Male & Female	1.022	1.020	1.024	< 0.0001

*Note:* RR reflects the relative risk per one‐year increase (time variable entered as continuous).

Abbreviations: CI, confidence interval; RR, relative risk.

#### 3.2.2 Age‐ and Sex‐Specific Temporal Trends

A significant interaction was observed between year and age group (*p* for interaction < 0.0001), indicating that temporal trends in appendectomy rates differed across age categories (Figure 3). Age‐stratified analyses revealed distinct temporal patterns with notable sex disparities, particularly in middle‐aged and older adults.

**FIGURE 3:**
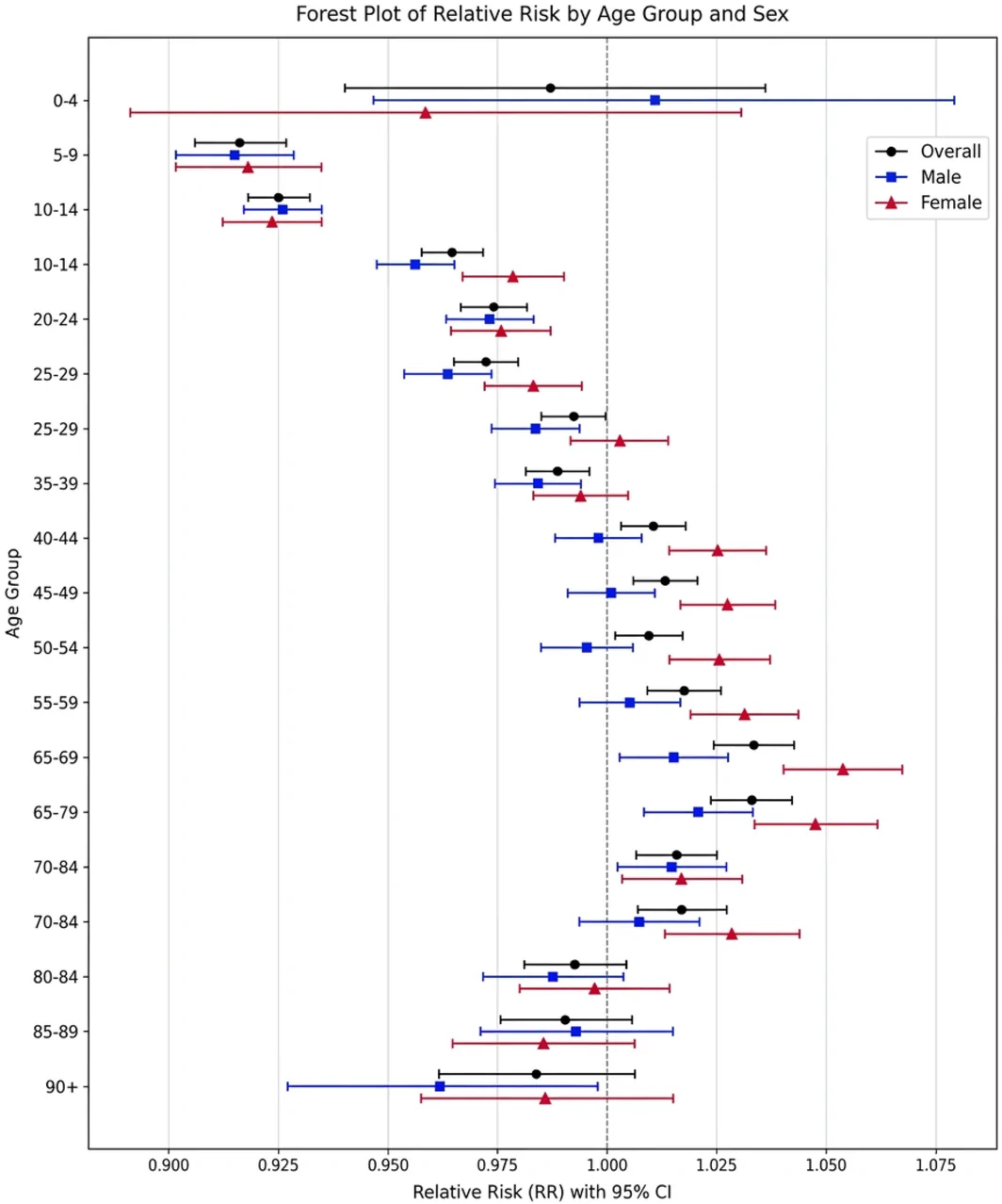
Annual RRs for appendectomy according to age group and sex, derived from Poisson regression models. RRs represent the relative change in the appendectomy rate associated with a 1‐year increase in fiscal year. An RR < 1 indicates an annual decrease in the appendectomy rate, whereas an RR > 1 indicates an annual increase. Results are shown for the overall population, men, and women. Horizontal lines indicate 95% confidence intervals. RR, relative risk.

Among children, adolescents, and young adults, appendectomy rates generally declined in both sexes. In the overall population, the most pronounced annual reductions were observed among individuals aged 5–29 years, with annual RRs ranging from 0.916 to 0.974 (all *p* < 0.0001), whereas the decline was less pronounced among those aged 30–39 years. Sex‐specific analyses showed that among men, appendectomy rates decreased significantly among those aged 5–39 years (RRs = 0.914–0.984). Among women, rates similarly declined from 5 to 29 years of age, whereas those aged 30–39 years exhibited a plateau with no statistically significant temporal change. In middle‐aged and older adults, temporal trends diverged substantially according to sex. Among men, appendectomy rates remained largely stable from 40 to 59 years of age, followed by modest but statistically significant increases at 60–74 years (RR = 1.015–1.021). Conversely, women exhibited a continuous, statistically significant annual increase from 40 to 79 years of age. These increases were particularly pronounced among women, especially at 60–69 years of age, reaching prominent peaks at 60–64 years (RR = 1.054, 95% CI: 1.040–1.067, *p* < 0.0001) and 65–69 years (RR = 1.047, 95% CI: 1.034–1.062, *p* < 0.0001). Among women aged ≥ 65 years, the number of appendectomies increased from 5935 in 2018 to 6744 in 2023, corresponding to an increase of 809 procedures (13.6%). The corresponding appendectomy rate increased from 29.79 to 32.88 per 100,000 person‐years during the same period. In the oldest age categories (≥ 80 years), appendectomy rates generally showed no significant temporal change in either sex, with trends attenuating toward unity and exhibiting wider CIs. To further assess whether the observed temporal patterns were dependent on a linear fiscal‐year assumption, a sensitivity analysis was performed in which fiscal year was treated as a categorical variable, with 2018 as the reference. Appendectomy rates among patients aged < 20 years showed a progressive decline from 2020 onward, whereas rates among those aged 20–64 years remained relatively stable overall, with modest fluctuations across years. Among patients aged ≥ 65 years, rates were also largely stable through 2022, followed by an increase in 2023 (Table S1).

### 3.3 Seasonal and Meteorological Analysis

Monthly nationwide appendectomies demonstrated significant seasonal variation (*p* for seasonality < 0.0001), with higher procedure numbers during Summer and lower numbers during Winter (Figure 4). The mean monthly procedure counts were 4319.8 (SD; 176.2), 4966.4 (SD; 299.4), 4429.8 (SD 319.6), and 3887.3 (SD 262.8) in Spring, Summer, Autumn, and Winter, respectively.

**FIGURE 4:**
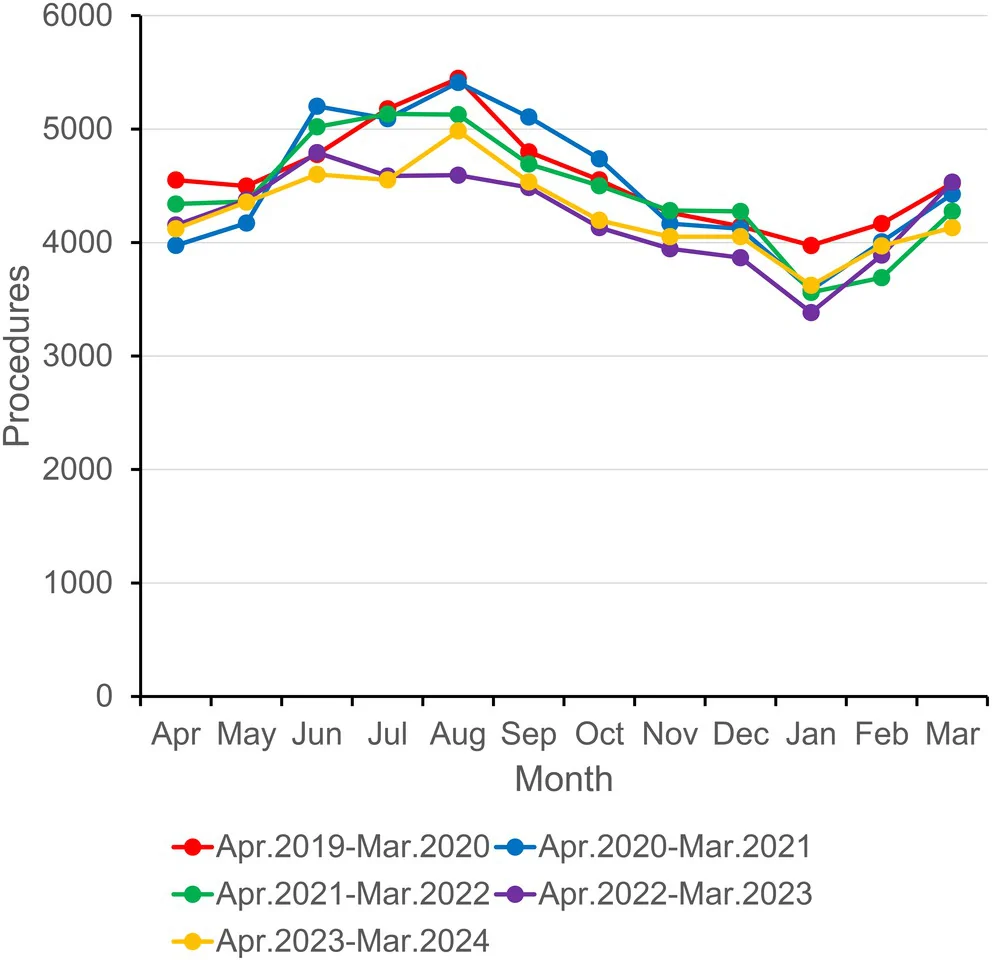
Monthly national appendectomies in 12 months between April 2019 and March 2024.

Pearson correlation analyses using the 30 day‐standardized monthly appendectomy counts demonstrated positive correlations between mean monthly temperature and appendectomy volume across all five representative cities (Figure 5). Correlation coefficients ranged from *r* = 0.837 in Sapporo to *r* = 0.845 in Nagoya. Barometric pressure showed inverse correlations with appendectomy volume, ranging from *r* = −0.431 in Sapporo to *r* = −0.811 in Fukuoka, whereas relative humidity showed moderate to strong positive correlations, ranging from *r* = 0.319 in Sapporo to *r* = 0.810 in Tokyo. Daylight hours (*r* = −0.303–0.561) and precipitation (*r* = −0.144–0.593) showed weaker and geographically inconsistent correlations (Table S2). These correlation analyses were considered exploratory.

**FIGURE 5:**
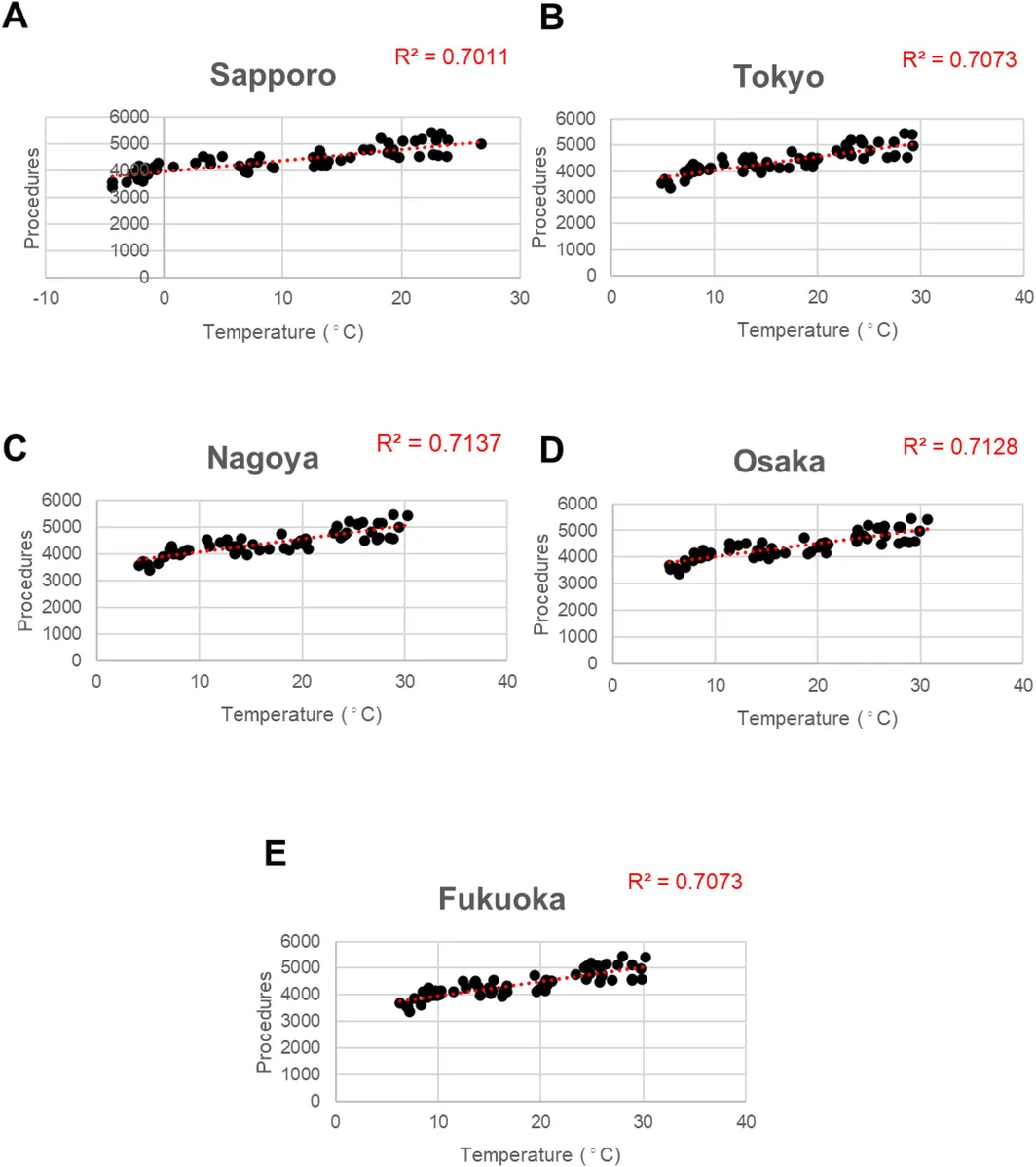
Scatter plots show correlations between monthly mean temperature data from (A) Sapporo, (B) Tokyo, (C) Nagoya, (D) Osaka, and (E) Fukuoka and their corresponding nationwide monthly appendectomy counts (y‐axis; standardized to a 30‐day basis) between April 2019 and March 2024. Each data point represents 1 month. City‐specific monthly mean temperature data were analyzed in relation to the same nationwide monthly appendectomy counts in each of the five models.

In the time‐series Poisson regression analysis, using the actual monthly number of appendectomies as the outcome and person‐time as an offset, mean monthly temperature was positively associated with monthly appendectomy volume after adjustment for calendar time and seasonal variation (Table 2). The association was consistent across the five city‐specific meteorological datasets, with relative risks ranging from 1.012 to 1.014 per 1°C increase in mean monthly temperature; all associations were statistically significant (*p* = 0.0027–0.0136).

**TABLE 2:** Association between monthly mean temperature and appendectomy rates estimated by time‐series Poisson regression.

City	RR	95% CI (low)	95% CI (high)	*p*
Sapporo	1.012	1.003	1.022	0.0136
Tokyo	1.014	1.005	1.023	0.0027
Nagoya	1.013	1.004	1.023	0.0060
Osaka	1.014	1.004	1.025	0.0076
Fukuoka	1.014	1.004	1.025	0.0071

*Note:* RR reflects the relative risk associated with a 1°C increase in monthly mean temperature.

Abbreviations: CI, confidence interval; RR, relative risk.

## 4 Discussion

This nationwide population‐based study provided a comprehensive overview of contemporary appendectomy epidemiology in Japan using the NDB Open Data. Although the nationwide age‐adjusted appendectomy rate showed only a slight decline from 2018 to 2023, the magnitude of this overall change was modest despite its statistical significance, and age‐stratified analyses revealed a considerable demographic heterogeneity that was completely obscured by the overall estimate. Appendectomy rates declined substantially among children and adolescents but increased progressively among older women. Concurrently, LA has become the predominant operative approach, and the appendectomy volume has demonstrated consistent seasonal variations that are positively associated with ambient temperature.

Notably, this study found that a relatively stable nationwide appendectomy rate concealed opposing temporal trends across age groups. Although previous epidemiological studies and global burden estimates have provided valuable insights into the overall disease burden and broad epidemiological trends [2, 17, 18, 19], the reliance on aggregated nationwide summary metrics can easily obscure subtle opposing temporal shifts across specific age groups. By incorporating age‐specific population‐adjusted analyses in a rapidly aging society, this study demonstrated that declining appendectomy rates among younger populations occur simultaneously with increasing rates among older adults. These findings suggest that population aging is progressively reshaping the demographic profile of patients undergoing surgery, while the observed increases in age‐specific appendectomy rates among older women represent a distinct phenomenon that cannot be explained by population aging alone. Consequently, age‐stratified analyses provide critical information that cannot be obtained using nationwide summary estimates alone.

The significant reduction in appendectomies in the younger population is likely multifactorial. Increasing acceptance of NOM for selected patients with uncomplicated appendicitis may have reduced the need for surgical intervention in younger individuals [20], whereas improvements in diagnostic imaging may have reduced unnecessary appendectomies through more accurate diagnosis [21]. Broader changes in environmental exposures [22], dietary habits [23], gut microbiota [24], and healthcare‐seeking behavior may have contributed to the observed decline in appendectomy rates. Shifts in healthcare use and hospital avoidance during and after the COVID‐19 pandemic have been shown to influence appendectomy rates across OECD member nations [25], suggesting that pandemic‐related behavioral changes may have contributed to the decline in surgical interventions. Although the relative contribution of these factors cannot be determined from this dataset, the consistent decrease observed across the pediatric and adolescent age groups indicates a clear age‐specific decline in appendectomy rates. However, because patients successfully managed with NOM and other non‐surgical strategies cannot be identified in the NDB Open Data, these findings should not be interpreted as evidence of a corresponding decline in the incidence of acute appendicitis.

Conversely, appendectomy rates have increased progressively among older women. Because these analyses were based on age‐specific population rates, this increase cannot be explained by population aging alone and represents a distinct increase in appendectomy rates within these age groups. Improvements in perioperative care, anesthetic management, and minimally invasive surgery have increased the feasibility of emergency surgery for elderly patients who may have previously been considered high‐risk candidates for operative treatment [26]. Advances in diagnostic imaging may facilitate earlier diagnoses in older patients who often present with atypical clinical manifestations [1, 5]. However, the substantially greater increase observed among women indicates that additional biological, demographic, and healthcare‐related factors may be contributing factors. The mechanisms underlying this distinctive sex‐specific trend remain unclear, necessitating investigation using patient‐level clinical data.

Another notable finding was the continued nationwide transition from OA to LA. By the end of the study period, LA accounted for most appendectomies performed nationwide, confirming its position as the standard operative approach in contemporary practice. This transition may reflect the accumulated surgical experience, improvements in laparoscopic technology, and increased confidence in minimally invasive emergency surgery [5]. Although the operative approach was not the primary focus of this study, the near‐complete replacement of an OA represents an important change in contemporary emergency surgical practice.

Consistent seasonality in the appendectomy volume was observed, with the number of procedures peaking during Summer. A recent nationwide NDB‐based study of rectal prolapse surgery in Japan also reported seasonal variation using a similar analytical approach, although the seasonal pattern differed from that observed in the present study [27]. Among all the meteorological variables evaluated, ambient temperature demonstrated the strongest and most consistent association with the nationwide monthly appendectomy volume across the five city‐specific meteorological datasets. Although the underlying biological mechanisms remain unclear, several explanations have been proposed, including dehydration, alterations in gastrointestinal physiology, infectious factors, and seasonal behavioral changes [6, 28]. Because this analysis was ecologically designed, these findings should be interpreted as population‐level associations. Although meteorological data from five cities were used to examine the consistency of the temperature‐related pattern across different locations, the same nationwide appendectomy counts were used for all five cities; therefore, these analyses do not represent independent regional replications. Accordingly, the geographic consistency of the association should be interpreted with caution.

These findings have important implications for the healthcare systems in aging societies. Although the overall nationwide appendectomy burden remained relatively stable, the demographic profile of patients undergoing appendectomy changed substantially, with increasing rates among middle‐aged and older women. Thus, the relatively small change in the overall appendectomy rate should not obscure shifts in the populations requiring emergency surgical care. Older patients frequently present with greater frailty, multiple comorbidities, and more complicated diseases, which potentially increase the demand for perioperative management and emergency surgical services [29]. Furthermore, the recognition of predictable seasonal fluctuations in appendectomy volume may assist hospitals in anticipating variations in the emergency operative workload. As Japan continues to experience rapid population aging, demographic changes may predict future trends in the epidemiology of appendectomies in other aging countries.

This study had several important strengths. The NDB provides near‐complete nationwide coverage under Japan's universal healthcare system, minimizing selection bias and enabling a comprehensive assessment of nationwide temporal trends. Furthermore, the simultaneous evaluation of demographic characteristics, operative approaches, and meteorological variables provides a broad overview of contemporary appendectomy epidemiology. Importantly, age‐specific Poisson regression analyses identified substantial demographic heterogeneity that was completely masked by nationwide summary estimates, highlighting the importance of demographic stratification in population‐based surgical studies.

This study had several limitations. First, the NDB Open Data comprised aggregated administrative data and excluded patient‐level clinical information, such as disease severity, laboratory findings, imaging results, pathological diagnosis, and postoperative outcomes. Consequently, the NDB Open Data do not allow differentiation between emergency appendectomy and interval appendectomy following initial NOM, and appendectomies performed for uncomplicated and complicated appendicitis were analyzed together. Furthermore, because the present analysis was based on procedures recorded under K718 or K718‐2, cases of appendicitis requiring extended resection beyond appendectomy, such as cecectomy or ileocecal resection, may not have been captured when these procedures were recorded under other surgical codes. Second, patients successfully treated with NOM could not be identified; therefore, changes in appendectomy rates should not be interpreted as direct changes in the incidence of appendicitis. Third, the exclusion of categories containing < 10 procedures may have influenced the estimates in the subgroups with low procedure counts, although the effect on nationwide trends is likely minimal. Fourth, because the study period encompassed the COVID‐19 pandemic, pandemic‐related changes in healthcare‐seeking behavior and access to emergency care, and treatment strategies, including the use of NOM, may have affected temporal trends in appendectomy rates, although the sensitivity analysis using fiscal year as a categorical variable showed broadly consistent age‐specific patterns. Finally, the ecological design of the meteorological analysis precludes causal inferences between environmental factors and the occurrence of appendectomies. Region‐specific linkage between appendectomy counts and corresponding meteorological data was not feasible with the NDB Open Data; therefore, the five‐city analysis relied on the same nationwide monthly appendectomy counts and did not constitute independent regional replication.

Overall, this study demonstrated that stable nationwide appendectomy rates conceal substantial age‐specific demographic changes. Although appendectomy rates have declined among younger populations, they have increased among older women, resulting in substantial changes in the demographic profile of patients undergoing appendectomy in Japan. Along with the nationwide transition toward laparoscopic surgery and consistent temperature‐associated seasonal variations, these findings highlight the importance of age‐stratified analyses for understanding nationwide surgical epidemiology and may contribute to future healthcare planning in aging societies. Because changes in appendectomy rates may reflect both changes in disease occurrence and changes in treatment strategies, including NOM, these findings should not be interpreted as direct evidence of changes in the incidence of acute appendicitis.

## Author Contributions

**Shutaro Sumiyoshi:** conceptualization, investigation, visualization, writing – original draft, formal analysis. **Masamitsu Kido:** conceptualization, investigation, writing – review and editing, methodology. **Tomohiro Arita:** supervision, writing – review and editing. **Naosuke Nakamichi:** writing – review and editing. **Kento Kurashima:** writing – review and editing. **Kenji Nanishi:** writing – review and editing. **Jun Kiuchi:** writing – review and editing. **Hiroki Shimizu:** writing – review and editing. **Kengo Yoshii:** formal analysis, validation, writing – review and editing. **Atsushi Shiozaki:** supervision, writing – review and editing.

## Funding

The authors have nothing to report.

## Ethics Statement

This study used publicly available, anonymized, and aggregated data from the NDB Open Data; therefore, institutional review board approval and informed consent were not required under the Ethical Guidelines for Life Science and Medical Research Involving Human Subjects.

## Conflicts of Interest

The authors declare no conflicts of interest.

## Supporting information


**Table S1:** Sensitivity analysis of temporal trends in appendectomy rates using fiscal year as a categorical variable.
**Table S2:** Correlation between monthly national appendectomy and meteorological data across the five major cities in Japan.

## Data Availability

The data analyzed in this study are publicly available from the National Database of Health Insurance Claims and Specific Health Checkups of Japan (NDB) Open Data and the official statistics portal of Japan (e‐Stat).
