# Supplementary material for: Age‐Specific Temporal Trends and Climate‐Associated Seasonality of Appendectomy in Japan: A Nationwide Population‐Based Study Using the National Database
Source: Ann Gastroenterol Surg. 2026 Oct 30:10.1002/ags3.70305. Online ahead of print. doi: 10.1002/ags3.70305 (PMC13720862; doi:10.1002/ags3.70305)
Supplement: Supplementary file 1 [file AGS3-9999-0-s001.docx]

**Supplementary Table 1.** Sensitivity analysis of temporal trends in appendectomy rates using fiscal year as a categorical variable

| **Year** | **Overall** |  | **<20 years** |  | **20–64 years** |  | **≥65 years** |  |
| --- | --- | --- | --- | --- | --- | --- | --- | --- |
|  | **RR (95% CI)** | **P-value** | **RR (95% CI)** | **P-value** | **RR (95% CI)** | **P-value** | **RR (95% CI)** | **P-value** |
| **2018** | 1.000 (Ref) | — | 1.000 (Ref) | — | 1.000 (Ref) | — | 1.000 (Ref) | — |
| **2019** | 1.016 (1.005–1.028) | 0.0066 | 0.984 (0.959–1.010) | 0.2359 | 1.035 (1.019–1.051) | <0.0001 | 1.003 (0.976–1.030) | 0.8503 |
| **2020** | 0.998 (0.986–1.010) | 0.7311 | 0.907 (0.883–0.931) | <0.0001 | 1.038 (1.022–1.054) | <0.0001 | 0.978 (0.952–1.006) | 0.1180 |
| **2021** | 0.990 (0.978–1.001) | 0.0857 | 0.880 (0.857–0.904) | <0.0001 | 1.023 (1.007–1.038) | 0.0037 | 1.027 (1.000–1.055) | 0.0519 |
| **2022** | 0.946 (0.935–0.958) | <0.0001 | 0.785 (0.764–0.808) | <0.0001 | 0.986 (0.971–1.002) | 0.0776 | 1.012 (0.985–1.040) | 0.3990 |
| **2023** | 0.976 (0.965–0.988) | <0.0001 | 0.776 (0.754–0.798) | <0.0001 | 1.025 (1.009–1.040) | 0.0016 | 1.058 (1.030–1.086) | <0.0001 |

RRs were estimated using Poisson regression with calendar year treated as a categorical variable and 2018 as the reference year, with the logarithm of the corresponding population as an offset.

**Supplementary Table 2.** Correlation between monthly national appendectomy and meteorological data across the five major cities in Japan.

| City | Temperature, °C | | Barometric pressure, hPa | | Humidity, % | | Daylight hours, h | | Precipitation, mm | | Location |
| --- | --- | --- | --- | --- | --- | --- | --- | --- | --- | --- | --- |
|  | r | P-value | r | P-value | r | P-value | r | P-value | r | P-value |  |
| **Sapporo** | 0.837 | < 0.001 | -0.431 | 0.001 | 0.319 | 0.013 | 0.561 | < 0.001 | -0.144 | 0.272 | 43° 3′ 43.49″ N,  141° 21′ 15.8″ E |
| **Tokyo** | 0.841 | < 0.001 | -0.650 | < 0.001 | 0.810 | < 0.001 | -0.303 | 0.019 | 0.455 | < 0.001 | 35° 40′ 52″ N,  139° 46′ 0″ E |
| **Nagoya** | 0.845 | < 0.001 | -0.762 | < 0.001 | 0.475 | < 0.001 | -0.100 | 0.447 | 0.579 | < 0.001 | 35° 10′ 53.04″ N, 136° 54′ 23.04″ E |
| **Osaka** | 0.844 | < 0.001 | -0.781 | < 0.001 | 0.624 | < 0.001 | 0.143 | 0.274 | 0.593 | < 0.001 | 34° 41′ 37.5″ N,  135° 30′ 7.6″ E |
| **Fukuoka** | 0.841 | < 0.001 | -0.811 | < 0.001 | 0.637 | < 0.001 | 0.314 | 0.014 | 0.521 | < 0.001 | 33° 35′ 24″ N,  130° 24′ 6.12″ E |

Values are presented as Pearson’s correlation coefficient (r), with corresponding P values.

Available 60 monthly data were assessed from April 2019 to March 2024.
